# Senkyunolides: a promising natural compounds for the treatment of migraine headaches

**DOI:** 10.3389/fnut.2026.1750201

**Published:** 2026-04-10

**Authors:** Yu Long, Dan Li, Xuemin Jian, Zhi Yang, Ting Leng, Xilian Wang, Wanxue Zhang, Xinyun Ge, Nan Li, Yuan Yin, Xiaoan Li

**Affiliations:** 1 National Health Commission Key Laboratory of Nuclear Technology Medical Transformation, Mianyang Central Hospital, School of Medicine, University of Electronic Science and Technology of China, Mianyang, China; 2 School of Modern Chinese Medicine Industry, School of Pharmacy, Chengdu University of Traditional Chinese Medicine, Chengdu, China; 3 College of Life Sciences and Agri-forestry, Southwest University of Science and Technology, Mianyang, China

**Keywords:** migraine, pharmacokinetics, pharmacology, senkyunolides, stability

## Abstract

Migraine, ranked as the second most disabling neurological disorder globally, affects over 1 billion people, imposing a substantial worldwide burden. The complexity of its pathological mechanisms contributes to therapeutic limitations. While existing first-line drugs offer partial efficacy, their utility is often constrained by cardiovascular risks, medication-overuse headaches, low bioavailability, and high treatment costs, necessitating novel therapies that balance efficacy and safety. Notably, senkyunolides, core bioactive compounds derived from traditional anti-migraine herbs like *Ligusticum chuanxiong*, have emerged as promising candidates due to the role of multidimensional homeostasis in regulating neurovascular units (suppressing activation of the trigeminal vascular system, modulating pathological vascular alterations, modulating neurotransmitters and receptors, inhibiting inflammatory response, antioxidant stress and analgesic effects and improving blood-brain barrier integrity) and unique pharmacokinetic advantages (small-molecule structure enabling blood-brain barrier penetration, natural origin reducing hepatorenal toxicity risks). This review systematically analyzes senkyunolides' chemical diversity, extraction methodologies, and anti-migraine pharmacological mechanisms. It further evaluates innovative solutions addressing the critical clinical translation bottleneck of instability. Beyond providing theoretical and technical foundations for developing “multi-target, low-toxicity” anti-migraine drugs, this work deepens understanding of transforming natural products into precision medicines, establishing a new paradigm for efficient and safe therapeutics. Future research must prioritize resolving formulation stability issues and rigorously advancing the clinical translation pipeline.

## Graphical Abstract

**Figure:**
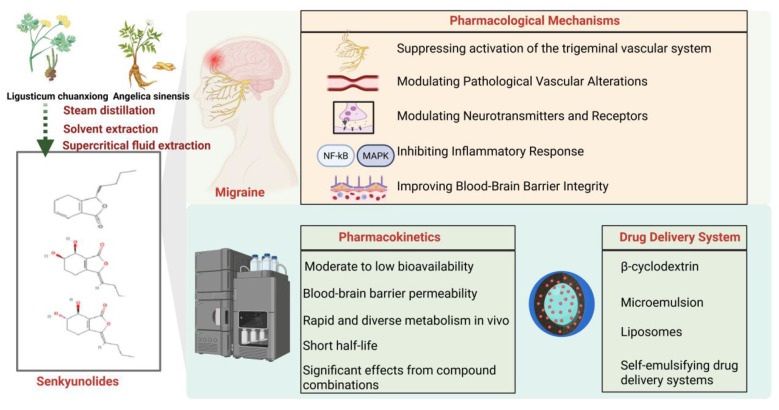
Senkyunolides, derived from Apiaceae plants such as Ligusticum chuanxiong and Angelica sinensis, exert anti-migraine effects through multi-target mechanisms including inhibition of trigeminal vascular system activation, regulation of vascular tone, anti-inflammatory and antioxidant actions, and protection of the blood-brain barrier. Despite the blood-brain barrier penetration advantage of senkyunolides, low bioavailability, short half-life, and chemical instability hinder clinical translation. Formulation strategies such as cyclodextrin inclusion complexes, micelles, and liposomes can effectively improve the pharmacokinetic properties and stability of senkyunolides. Created with BioRender.com.

## 1 Introduction

Migraine is a chronic neurovascular disorder, characterized primarily by moderate to severe pulsating headaches accompanied by primary vascular and neurological dysfunction (1). According to the Global Burden of Disease, Injuries, and Risk Factors Study, migraine ranks as the second leading cause of disability worldwide. The global prevalence of migraine has surged from 732.56 million cases in 1990 to 1.16 billion in 2021, currently affecting approximately 1 billion people globally. This condition imposes a particularly heavy burden on individuals under 50 years of age, representing a significant public health challenge (2). According to the International Classification of Headache Disorders, migraines are categorized into three primary types: migraine with aura, migraine without aura, and chronic migraine (3). Clinical manifestations in migraine patients include recurrent headache attacks characterized by unilateral location, pulsating quality, moderate or severe intensity, aggravation by routine physical activity, and association with nausea, vomiting, photophobia, and phonophobia (4). Currently, the diagnosis of migraine continues to rely principally on clinical criteria, although emerging research is actively pursuing specific biomarkers to more precisely characterize its underlying pathophysiology. At present, no disease-modifying therapy is available for migraine. Existing drugs primarily serve to alleviate symptoms rather than cure the condition. Given their inherent capacity for multi-component synergy, multi-target interventions, and favorable safety profiles, natural medicines have emerged as a pivotal direction in anti-migraine drug development. In contrast to conventional synthetic agents, natural medicines, derived from botanical and other natural sources, generally exhibit a reduced burden of side effects and are better suited for long-term therapeutic use. Their defining characteristics, which include complex pharmacologically active compositions, the ability to engage multiple therapeutic targets concurrently, low intrinsic toxicity, and wide availability, have attracted considerable scientific interest in recent years. As a result, natural medicines are increasingly regarded as viable alternatives or complements to single-target chemical pharmaceuticals.

Senkyunolides, a class of phthalide-type compounds, are naturally derived from Apiaceae plants such as *Ligusticum chuanxiong* (Chuanxiong), *Angelica sinensis* (Danggui), *Ligusticum sinense* (Gaoben), *Apium graveolens* (Celery), *Opopanax chironium, Lomatium californicum, Cnidium officinale, Cnidii Rhizoma, Levisticum officinale, Angelica acutiloba*, and *Peucedanum ostruthium* (5–10). This class of compounds encompasses multiple structural variants (e.g., senkyunolide A–S), which have attracted considerable scientific interest for their potential to alleviate migraine symptoms. Their putative mechanism of action involves the modulation of key neurotransmitters and inflammatory pathways central to migraine pathophysiology. Senkyunolides have attracted considerable scientific interest owing to their promising potential in alleviating migraine symptoms, primarily through the modulation of key neurotransmitters and inflammatory pathways involved in the disorder's pathophysiology. As key constituents in Chuanxiong (11), senkyunolide A represents the second most abundant compound after ligustilide, with concentrations surpassing those of ferulic acid and tetramethylpyrazine, underscoring its role as a critical bioactive component of this medicinal plant. Notably, although ligustilide itself exhibits limited stability and oral bioavailability, it undergoes efficient metabolic conversion into senkyunolides A and I (12, 13). In contrast, senkyunolides demonstrate superior lipophilicity and stability, enabling rapid absorption into the bloodstream and cerebrospinal fluid. Their ability to cross the blood-brain barrier (BBB) enhances bioavailability and contributes to their therapeutic efficacy against migraine and associated disorders (14).

This review focuses on the anti-migraine pharmacological mechanisms, pharmacokinetic characteristics, and formulation stability optimization strategies of senkyunolides. By integrating these perspectives, we aim to establish a theoretical foundation for their clinical translation and to promote the development of effective, well-tolerated natural therapeutics for migraine.

## 2 Historical treatment of migraine

Migraine, an ancient affliction with documented symptoms (e.g., unilateral throbbing pain, nausea, photophobia), traces its earliest descriptions to classical medical texts. The *Ebers Papyrus* (c. 1550 BCE) from ancient Egypt references ailments affecting “one side of the head”, often attributed to invasive pathogenic winds. In Epidemics, Hippocrates (460–370 BCE) provided the first clear description of migraine with aura, linking it to vascular dilation and recording visual premonitory symptoms (“scintillating scotomas”) that precede headache attacks. He also introduced the term hemicrania (literally “half-head”). Later, Galen (129–216 CE) advanced the “humoral imbalance” theory, proposing that attacks resulted from an accumulation of melancholic bile or overheated blood (15). Subsequent anatomical investigations gradually shifted focus toward the cranial vasculature and neural pathways. Within the framework of traditional Chinese medicine, the Huangdi Neijing (c. 200 BCE) conceptualized a migraine-like disorder under the syndrome termed jueni toutong (“reversal headache with cold limbs”), thereby establishing early foundational theories for its diagnosis and treatment (16).

Senkyunolides, a class of phthalide compounds isolated from *Ligusticum chuanxiong* and related plants, demonstrate multifaceted pharmacological activities including anti-inflammatory, analgesic, and microcirculation-enhancing effects. Historically, plants containing senkyunolides have been extensively documented for migraine treatment across medical traditions. In China, the Shennong Bencaojing (c. 200 CE) provided the earliest pharmacopeia record of Chuanxiong for headache management, specifically noting its efficacy against migraine-like headaches. This canonical text established its therapeutic role in traditional Chinese medicine for managing cephalic disorders, particularly those attributed to pathogenic wind invasion (17). Chuanxiong Chatiao San, first recorded in the Song Dynasty formulary Taiping Huimin Hejiju Fang (c. 1241 CE), is composed of eight herbs: *Ligusticum chuanxiong, Mentha haplocalyx, Asarum sieboldii, Schizonepeta tenuifolia, Saposhnikovia divaricata, Angelica dahurica, Notopterygium incisum*, and *Glycyrrhiza uralensis*. This classical formula has been historically prescribed for wind-pathogen headaches, migrainous or diffuse cephalgia, and febrile symptoms induced by wind-cold invasion (18). Danxi Xinfa (1347 CE) proposed medication principles for migraine, indicating that Chuanxiong and Danggui were essential treatments for migraines caused by blood deficiency. The Bencao Gangmu (1596 CE) mentioned that Chuanxiong and Danggui were used in ancient times to treat headaches (19). The Qing Dynasty text Bencao Beiyao (1683 CE) also recorded Danggui's property of “ascending to treat headaches”, noting that its combination with Chuanxiong enhanced therapeutic efficacy (20). In Japanese traditional medicine, Chuanxiong is used to treat diverse conditions. Ishinpo (984 CE), the oldest extant medical text in Japan, recorded multiple applications of Chuanxiong, including its use for headache management (21). In Korea, the Joseon-era medical work described in its 1,433 edition a formula called “Chuanxiong San”, composed of Chuanxiong, Asarum, and Angelica dahurica, which was employed to treat unilateral or diffuse headaches (22). Furthermore, the Dongui Bogam (1613) included an entry under “Head Section·Migraine” that recorded the use of “Danggui Wine” for migraines attributed to blood deficiency (23). In medieval Europe, where herbal medicine predominated, *Ligusticum chuanxiong* and *Angelica sinensis* (introduced from China) were used alongside native European herbs such as valerian and chamomile to relieve headache symptoms. This historical evidence demonstrated that Chuanxiong and Danggui served as core therapeutic agents in long-standing migraine management (24). Modern research confirms that their active constituent, the senkyunolides, exhibit neuroprotective, anti-inflammatory, vascular homeostasis-regulating, and analgesic properties—mechanistically explaining their traditional efficacy and highlighting their significant anti-migraine potential.

## 3 Classification, extraction, and isolation of senkyunolides

Senkyunolides are chemically defined as lactones, characterized by a fused γ-lactone and benzene bicyclic core. Their notable structural diversity stems from the high susceptibility of the benzene ring and side chains to various substitutions and modifications. Based on their structural architecture, senkyunolides are primarily categorized into two classes: simple phthalides (SP) and dimeric phthalides (DP) (25), as shown in Table 1. Senkyunolides represent the most abundant group of phthalide compounds in Chuanxiong after ligustilide (26), with senkyunolide A, followed by I and H, being the predominant forms (27). Beyond Chuanxiong, other botanical sources also contain significant amounts of these compounds: *Cnidium officinale* Makino is rich in senkyunolides A through J (A–J) (28), and *Meum athamanticum* yields isolable senkyunolides A, C, E, F, H, and I (29). Despite their wide structural diversity across species, pharmacological research has remained largely concentrated on senkyunolides A, H, and I. This focused investigation likely reflects the greater chemical instability of other analogs, which poses a challenge to their comprehensive study.

**Table 1:** Classification of senkyunolide compounds.

Compound	Source	Structural formula	Molecular formula	CAS no.	Classification (SP/DP)
Senkyunolide A	*Ligusticum chuanxiong* Hort.	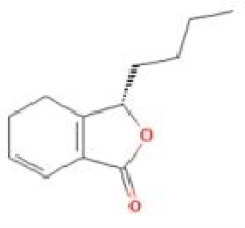	C_12_H_16_O_2_	63038-10-8	SP
Senkyunolide B	*Ligusticum chuanxiong* Hort.	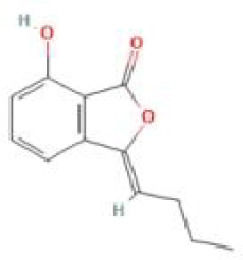	C_12_H_12_O_3_	93236-67-0	SP
Senkyunolide C	*Cnidium officinale*	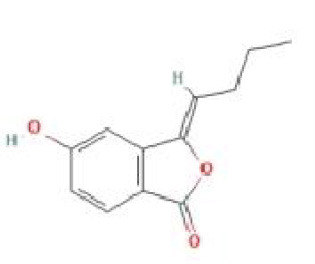	C_12_H_12_O_3_	91652-78-7	SP
Senkyunolide D	*Ligusticum chuanxiong* Hort.	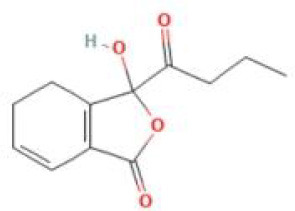	C_12_H_14_O_4_	94530-82-2	SP
Senkyunolide E	*Dicerothamnus rhinocerotis* and *Ligusticum chuanxiong* Hort.	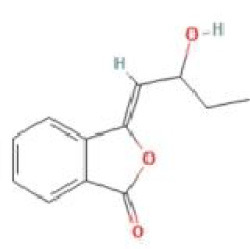	C_12_H_12_O_3_	94530-83-3	SP
Senkyunolide F	*Ligusticum chuanxiong* Hort.	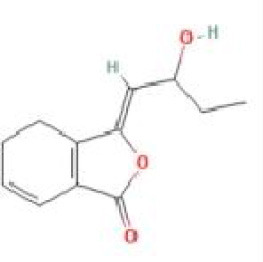	C_12_H_14_O_3_	94530-84-4	SP
Senkyunolide G	*Angelica sinensis* and *Echinacea angustifolia* DC.	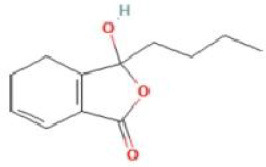	C_12_H_16_O_3_	94530-85-5	SP
Senkyunolide H	*Ligusticum chuanxiong* Hort	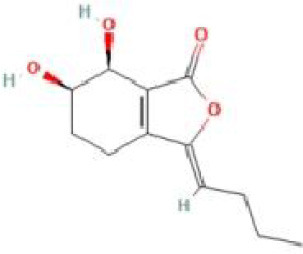	C_12_H16O4	94596-27-7	SP
Senkyunolide I	*Ligusticum chuanxiong* Hort. and *Cnidium officinale*	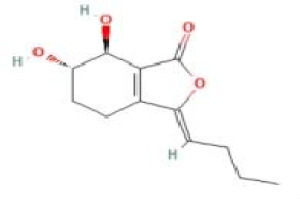	C_12_H_16_O_4_	94596-28-8	SP
Senkyunolide J	*Ligusticum chuanxiong* Hort. and *Apium graveolens* L.	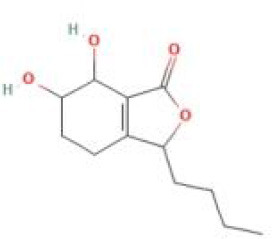	C_12_H_18_O_4_	94530-86-6	SP
Senkyunolide K	*Ligusticum chuanxiong* Hort. and *Ligusticum striatum* DC.	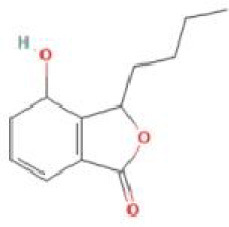	C_12_H_16_O_3_	114569-33-4	SP
Senkyunolide L	*Ligusticum chuanxiong* Hort.	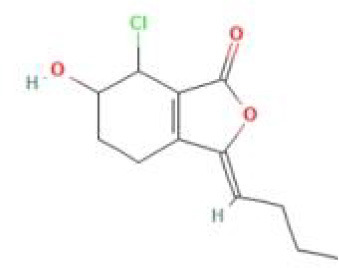	C_12_H_15_ClO_3_	114586-51-5	SP
Senkyunolide N	*Ligusticum striatum, Apium graveolens* and *Ligusticum chuanxiong* Hort.	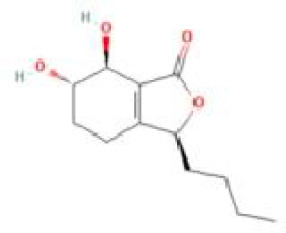	C_12_H_18_O_4_	140694-58-2	SP
Senkyunolide O	*Ligusticum chuanxiong* Hort.	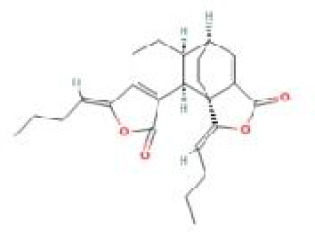	C_24_H_28_O_4_	142797-35-1	DP
Senkyunolide R	*Ligusticum striatum* and *Ligusticum chuanxiong*	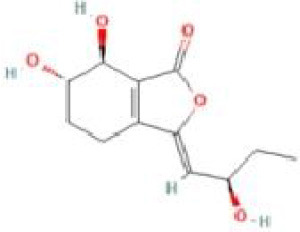	C_12_H_16_O_5_	172549-37-0	SP
Senkyunolide S	*Ligusticum striatum* and *Ligusticum chuanxiong*	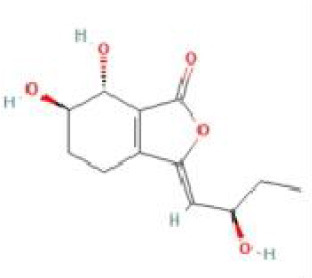	C_12_H_16_O_5_	172723-28-3	SP

Current extraction methodologies for senkyunolides primarily employ steam distillation, solvent extraction, and supercritical fluid extraction. Steam distillation, which employs water vapor to isolate volatile constituents from plant material, offers the advantages of operational simplicity and minimal equipment requirements. However, its utility is limited by relatively low extraction yields and the risk of thermal degradation or isomerization of the target lactones due to prolonged exposure to high temperatures. In contrast, supercritical carbon dioxide (CO_2_) extraction operates under mild conditions by utilizing the high solvating power of supercritical fluids. This method demonstrates superior extraction efficiency for senkyunolides while circumventing the thermal degradation associated with steam distillation. Nevertheless, its widespread industrial application is constrained by high capital costs, operational complexity, and significant overall expenses. Solvent reflux extraction, typically performed with organic solvents under controlled temperature, achieves higher yields through relatively mild processing. Ethanol is the most commonly employed solvent in current practice, followed by methanol and water. Studies have shown that pretreatment with cellulase can further enhance the dissolution of lactones during ethanol-based extraction of ligusticum components (30).

Following initial extraction, senkyunolides are typically purified via reversed-phase high-performance liquid chromatography (RP-HPLC). Xiaozhe Zhang et al. achieved rapid isolation of target compound senkyunolide-I and byproduct ferulic acid from *Ligusticum chuanxiong*'s 95% (v/v) ethanol extract using RP-HPLC with ammonium acetate as modifier, employing step gradient elution and resin column desalination, yielding purities exceeding 98% (31). Concurrently, Yun Wei et al. selected an optimized n-hexane-ethyl acetate-methanol-water (3:7:4:6, v/v) solvent system for countercurrent chromatography based on partition coefficients (k) and separation factors (α), utilizing the lower phase as mobile phase in head-to-tail elution mode, where 400 mg crude extract yielded pure senkyunolide-I (6.4 mg, 98%) and senkyunolide-H (1.7 mg, 93%) in a single run (32). Jianmin Cao optimized the extraction process by targeting the total content of four major lactones, establishing 75% ethanol (10 × volume) with double reflux at 60 °C followed by ethyl acetate extraction (11 ml/g crude herb) as optimal protocol; RP-HPLC quantification confirmed total lactone content (senkyunolide-H, senkyunolide-I, sedanonic acid lactone, and ligustilide) in the extract reached 24%−30% (33).

## 4 Anti-migraine mechanisms of senkyunolides

**Figure 1:**
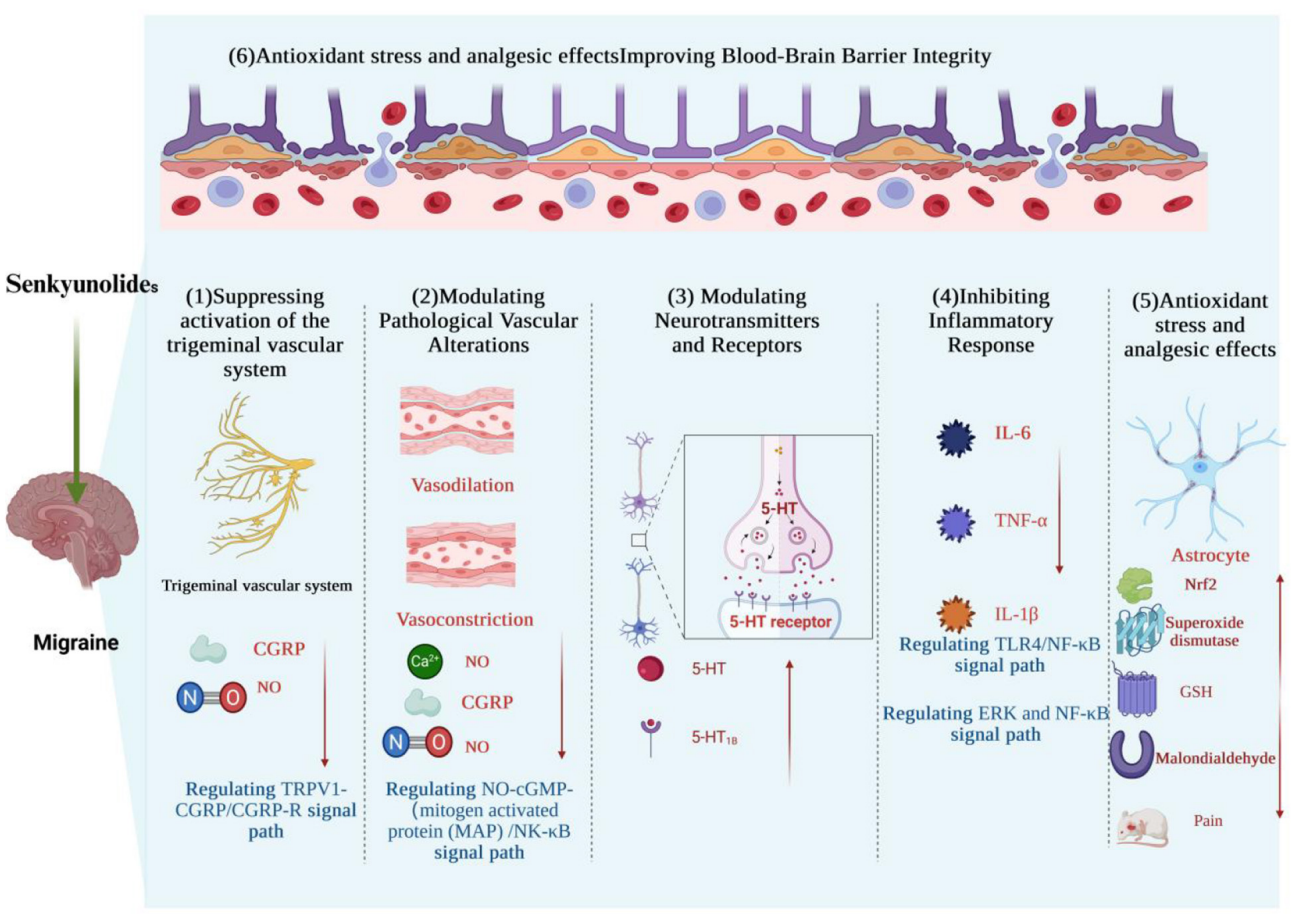
Pharmacological mechanisms of senkyunolides in ameliorating migraine. Created with BioRender.com.

As a complex neurological disorder, migraine pathogenesis involves multifaceted mechanisms centered on abnormal activation of the trigeminovascular system, which triggers the release of neuropeptides, subsequently inducing neurogenic inflammation and central sensitization; genetic factors (combined with environmental triggers) establish the pathological foundation, ultimately generating characteristic symptoms—unilateral pulsating headaches, photophobia, and nausea—through neurovascular-inflammatory cascades (34). The neurovascular unit is a functionally integrated complex comprising closely interconnected neurons, glial cells, vascular endothelial cells, and pericytes. In migraine, dysfunction in each component interacts and influences the others, forming a vicious cycle. During a migraine attack, inflammatory factors released upon activation of the trigeminovascular system induce oxidative stress, generating a large amount of ROS. This degrades tight junction proteins and damages pericytes, leading to disruption of BBB integrity. The increased barrier permeability allows peripheral inflammatory mediators to invade the perivascular space, which further activates neuroinflammation and releases more CGRP and ROS, creating a feedback loop that drives central sensitization. Mechanistically, senkyunolides share partial similarities with triptans, CGRP monoclonal antibodies, and ditans—for example, in inhibiting CGRP release. However, senkyunolides not only act on the aforementioned neurovascular targets but, more importantly, are involved in multidimensional homeostatic regulation of the neurovascular unit (by suppressing activation of the trigeminal vascular system, modulating pathological vascular alterations, modulating neurotransmitters and receptors, inhibiting inflammatory response, counteracting oxidative stress, producing analgesic effects, and improving BBB integrity) (Figure 1). Thereby, they exert multi-link modulation on the complex pathological network of migraine, which stands in sharp contrast to existing mainstream single-target or highly specific targeted drugs. The multi-mechanistic synergistic action of senkyunolides may offer a novel perspective for the prevention of chronic migraine.

### 4.1 Suppressing activation of the trigeminal vascular system

The central event in a migraine attack is the aberrant activation of the trigeminovascular system. Stimulation of dural blood vessels promotes the release of vasoactive neuropeptides, thereby inducing dural vasodilation, plasma protein extravasation, and mast cell degranulation. These processes contribute to neurogenic inflammation and pain sensitization. CGRP, the most potent vasodilatory peptide identified to date, is widely distributed in the trigeminal ganglion and perivascular nerve fibers of cerebral blood vessels (35–37). Therefore, inhibiting the excessive release of CGRP or blocking its receptors represents the most advanced therapeutic target for migraine treatment. Erenumab is the first monoclonal antibody targeting CGRP approved by the U.S. Food and Drug Administration. While Erenumab is safe and effective for long-term use, it may cause adverse reactions such as injection site reactions, constipation, and muscle spasms (38). In recent years, efforts have been dedicated to identifying natural CGRP inhibitors. Senkyunolide has been found to be a natural regulator of CGRP. Studies indicate that senkyunolide can effectively treat migraines by reducing protein content and downregulating CGRP mRNA expression in both serum and the cerebral cortex (39).

Studies have revealed that senkyunolide I could influence the levels CGRP in plasma and brain tissues of rats (40–42). Furthermore, additional research demonstrated that the ameliorative effect of senkyunolide I on migraines exhibited a dose-dependent trend. After intervention with a high dose of senkyunolide I (144 mg/kg), plasma levels of CGRP and nitric oxide (NO) in migraine model rats were significantly reduced (*P* < 0.05), while medium (72 mg/kg) and low doses (36 mg/kg) only decreased CGRP levels. This finding suggested that the CGRP and NO signaling pathways were critical targets through which senkyunolide I exerted its effects (43). Chen Ling compared the therapeutic effects of different fractions of *Ligusticum chuanxiong*—including ethyl acetate, petroleum ether, dichloromethane, and n-butanol fractions—on migraine model rats. It was found that the petroleum ether, ethyl acetate, and n-butanol fractions all reduced the frequency of migraine-related behaviors (head shaking, forelimb scratching, and hindlimb facial tapping) in the rats. These fractions also significantly decreased the levels of CGRP in the plasma of migraine model rats. Among them, the ethyl acetate fraction (which contains senkyunolide I, senkyunolide H, senlactone A, ligustilide, etc.) exhibited the strongest anti-migraine effect (44). Chuanxiong Rhizoma and Cyperi Rhizoma (CRCR) is an ancient classical formula composed of Chuanxiong and Xiangfu (*Cyperus rotundus*) in a 1:2 weight ratio, long used for migraine treatment. CRCR exerted significant therapeutic effects by increasing cerebral blood flow, reducing the expression of CGRP and c-Fos mRNA. Notably, after oral administration of CRCR, multiple active components—including senkyunolide A—were detected in the serum and cerebral cortex of rats, suggesting that senkyunolide A may be one of the key bioactive components underlying the anti-migraine efficacy of CRCR (39). Xiongmatang (XMT) is a classical traditional formula composed primarily of two medicinal herbs: Chuanxiong and Tianma (*Gastrodia elata*). The main chemical components of XMT include ligustilide, ferulic acid, senkyunolide, and gastrodin (45). Clinically, XMT has been demonstrated to significantly alleviate migraine-associated symptoms such as nausea, vomiting, and photophobia in patients. It reduced the frequency and shortens the duration of headache attacks. Moreover, XMT exerted therapeutic effects by decreasing serum levels of CGRP (46). Preclinical studies demonstrate that XMT extract ameliorates both behavioral deficits and pathological alterations in the trigeminal nerve of rats. Mechanistically, XMT extract downregulates the expression of key migraine-related mediators in the trigeminal system, as evidenced by reductions in the immunohistochemical staining, mRNA levels, and protein abundance of transient receptor potential vanilloid 1 (TRPV1), CGRP, calcitonin receptor-like receptor (CLR), and receptor activity-modifying protein 1. These effects are likely mediated through modulation of the TRPV1–CGRP/CLR signaling pathway (47). Furthermore, separate studies indicate that certain constituents of Chuanxiong, such as senkyunolide I, can enhance the central nervous system uptake of active components from Tianma (*Gastrodia elata*), slow their systemic and cerebral clearance, prolong their retention and half-life, and thereby improve overall bioavailability (48, 49).

### 4.2 Modulating pathological vascular alterations

Following the activation of the trigeminovascular system, the release of vasoactive substances can lead to pathological dilation or constriction of intracranial and extracranial blood vessels, thereby serving as a major contributor to the pulsatile pain characteristic of migraine. Migraine is fundamentally a neurological disorder in which vascular alterations arise as a consequence of dysfunctional neural regulation. While these vascular changes constitute a critical intermediate step and active component of the migraine attack cascade, they are generally viewed as secondary phenomena rather than the primary etiology. Cortical spreading depression (CSD), a slowly propagating wave of near-total neuronal depolarization in the cerebral cortex, is closely linked to migraine aura. CSD is initially characterized by a brief phase of reduced cerebral blood flow, termed the initial hypoperfusion phase (50–52). The wave of neuronal and glial depolarization during CSD drives a sharp increase in local metabolic demand. However, because the accompanying vasodilation is initially insufficient to match this demand, a transient period of relative ischemia ensues. This is followed by a subsequent hyperperfusion phase, characterized by a pronounced increase in cerebral blood flow. This reactive vasodilation is primarily mediated by neurovascular coupling: the heightened neuronal activity triggers the release of vasodilatory mediators to augment local blood supply and meet the elevated metabolic requirements (53, 54). Experimental studies have shown that a high dose of senkyunolide I significantly inhibits the wave amplitude of CSD induced by potassium chloride in rats. Inhibiting CSD can reduce the activation of the trigeminovascular system at its source (43). During the migraine aura or other specific conditions, cerebral blood vessels may experience sustained tonic contraction or spasm, resulting in compromised local perfusion. Senkyunolide can relax spasmed blood vessels by regulating ion channels and endothelial function, thereby restoring normal vascular tone and blood supply, improving circulation, and alleviating neurological symptoms caused by ischemia or hypoxia (55). Calcium channel blockers selectively inhibit slow calcium channels, reduce calcium influx, and exert vasodilatory and cerebral blood flow-improving effects (56). Shunaoxin pills (SNX) could dilate cerebral blood vessels and increase cerebral blood flow by regulating calcium-related signaling pathways and restoring calcium homeostasis. The vasodilatory effect of SNX appears to operate predominantly through endothelium-independent mechanisms. This process may involve multiple pathways, including the NO/cGMP cascade, the carbon monoxide/hydrogen sulfide system, blockade of calcium ion (Ca^2+^) channels, and inhibition of inositol trisphosphate receptor-mediated intracellular Ca^2+^ mobilization (57). By employing an activity-based screening platform that integrates ultra-performance liquid chromatography–quadrupole time-of-flight mass spectrometry with a dual-luciferase reporter calcium assay, Jin Zhang et al. identified senkyunolide I as an active component contributing to the bioactivity of SNX (58). In a separate study, senkyunolide I, senkyunolide A and ligustilide were recognized as potential calcium antagonists present in Suxiao Jiuxin Pill. Notably, senkyunolide A appears to exert its inhibitory effect primarily on ryanodine receptors and, to a lesser extent, on voltage-gated Ca^2+^ channels (59). Furthermore, senkyunolide A could relax isolated aortic vessels in model rats and inhibit vasoconstriction induced by prostaglandin E2, phenylephrine, serotonin, and other agents (60).

CGRP exerts its biological effects by binding to receptors on vascular endothelial cells and vascular smooth muscle. For instance, in vascular endothelial cells, it mediates the activation of nitric oxide synthase (NOS), leading to the production of NO. NO can directly stimulate vascular endothelium to induce vasodilation, while also acting on vascular smooth muscle cells to promote the accumulation of cyclic guanosine monophosphate (cGMP)—produced by guanylate cyclase—and activate ion channels. This process enhances potassium ion (K^+^) influx and Ca^2+^ efflux, collectively contributing to vasodilation. Furthermore, while promoting vasodilation, NO also functions as a neurotransmitter, participating in the regulation of CGRP synthesis and release in trigeminal ganglion neurons. This facilitates pain signal transmission and maintenance. Under the synergistic interaction of CGRP and NO, sustained dilation of intracranial and extracranial blood vessels occurs, promoting central sensitization and ultimately leading to the development of migraine (2). As noted above, senkyunolides can inhibit CGRP release and thereby counteract pathological vasodilation. NO participates in pain transmission, hyperalgesia, chronic pain, inflammation, and central sensitization, primarily through a cGMP-dependent mechanism (61–63). NO plays a pivotal role in the pathogenesis of migraine as an endothelium-derived vasodilator, an effect that is likely mediated through the NO–cGMP–mitogen-activated protein kinase (MAPK)/nuclear factor kappa-B (NF-κB) signaling pathway (64). Studies have shown that senkyunolide I could reduce the levels of NO and NOS in both plasma and brain tissues (41). Furthermore, senkyunolide I inhibited the NF-κB pathway and modulates the levels of NO and CGRP, both of which were closely associated with vasodilation during migraine attacks (65). Collectively, these findings indicate that the vascular effects of senkyunolides extend beyond mere vasoconstriction or vasodilation, and instead reflect a normalization of pathological vascular dysfunction.

### 4.3 Modulating neurotransmitters and receptors

The monoamine neurotransmitter system plays a significant role in the pathophysiology of migraine, contributing not only to pain modulation but also to common migraine-associated symptoms such as nausea, vomiting, and mood disorders. 5-Hydroxytryptamine (5-HT) is one of the therapeutic targets for migraine and has been identified as a vasoconstrictor present in the blood. Low plasma levels of 5-HT are believed to play a role in migraine pathophysiology (66–68). The regulation of monoamine neurotransmitters by senkyunolide I is an important mechanism through which it alleviates migraine and its associated symptoms. In a rat model of migraine induced by nitroglycerin, the levels of three monoamine neurotransmitters—5-HT, its precursor 5-HTP, and its metabolite 5-HIAA—were all reduced. The conversion ratio of 5-HTP to 5-HT was reduced, while the conversion ratio of 5-HT to 5-HIAA was elevated (40). Senkyunolide I has been shown to regulate monoamine neurotransmitter levels in both plasma and brain tissues of nitroglycerin-induced migraine model rats, with peak effects observed at 4 h after administration (40). Experimental evidence indicated that senkyunolide I could modulate key neurochemical pathways involved in migraine pathophysiology. Oral administration of senkyunolide I has been shown to concurrently regulate plasma and brain tissue levels of β-EP and ET-1, and to influence both the concentration and turnover rate of the monoamine neurotransmitter 5-HT in the brain (41, 42). β-EP, which can activate opioid receptors in the central nervous system, thereby produce potent analgesic effects.

With in-depth research on 5-HT receptor subtypes, receptors such as 5-HT_1B_, 5-HT_1D_, 5-HT_2C_, and 5-HT_1F_ have been found to play important roles in the pathophysiology of migraine. Among them, agonists of 5-HT_1B/1D_ receptors have become first-line drugs for the treatment of acute migraine attacks (69). The volatile oil derived from Chuanxiong has demonstrated efficacy in alleviating migraine-like symptoms in nitroglycerin-induced rat models. This effect is associated with elevated plasma 5-HT levels, increased mean optical density of 5-HT_1B_ receptor-positive cells, and upregulation of 5-HT_1B_ protein expression in the periaqueductal gray region (70). Activation of 5-HT_1B_ receptors can inhibit trigeminovascular excitability and reduces the release of inflammatory mediators, constituting a key mechanism of action for triptan drugs (71). This mechanistic alignment suggests that senkyunolides may produce their therapeutic effects through a similar pathway.

Research found that CRCR significantly increased cerebral blood flow in nitroglycerin-induced migraine model rats, reduced levels of ET-1, GABA and elevated levels of 5-HT, 5-HIAA, and β-EP (39). Furthermore, a study on CRCR dropping pills demonstrated that this compound formula could increase the content of 5-HT and β-EP in the hypothalamus of migraine model rats, while reducing plasma levels of ET-1. This bidirectional regulation of both pro-algesic and analgesic mediators epitomizes the multi-target and systemic regulatory approach characteristic of traditional Chinese medicine compound formulations. Quantitative analysis via HPLC further confirmed that CRCR dropping pills contain senkyunolide I (2.88%) and senkyunolide A (2.17%) (72). Beyond the pairing of *Chuanxiong Rhizoma* and *Cyperi Rhizoma*, the synergistic interaction between senkyunolides from Chuanxiong and gastrodin from Tianma has been shown to markedly reduce the frequency and severity of headache episodes in migraine patients, while effectively modulating serum concentrations of 5-HT and other relevant biomarkers.

### 4.4 Inhibiting inflammatory response

Neurogenic inflammation serves as a critical amplifier within the migraine pathological network, with NF-κB serving as a key regulator of this inflammatory process. During a migraine attack, various inflammatory factors such as tumor necrosis factor-α (TNF-α) and interleukin-1β (IL-1β) are released, which can activate NF-κB (73, 74). Upon activation, NF-κB translocates to the nucleus, where it drives the transcription of pro-inflammatory genes—such as iNOS and cyclooxygenase-2 (COX-2)—and orchestrates the expression of numerous inflammatory mediators, thereby initiating neurogenic inflammation (75). This cascade results in vasodilation, elevated vascular permeability, plasma protein extravasation, and tissue edema, collectively contributing to pain. Moreover, during migraine attacks, NF-κB activation further amplifies the release of vasoactive substances, which in turn promote vasodilation and enhance vascular permeability, thereby exacerbating headache symptoms (76). Menglin et al. utilized an integrated approach combining ultra-performance liquid chromatography/quadrupole-time-of-flight analysis, an NF-κB dual-luciferase reporter assay, and spectrum–effect correlation screening to systematically identify and characterize NF-κB-inhibitory components in Angelica. Their work confirmed that chlorogenic acid, senkyunolide I, and Z-ligustilide constitute the principal active constituents responsible for the anti-inflammatory activity of this medicinal plant (60, 77).

Ningning et al. conducted a spectrum–effect correlation analysis to evaluate the anti-inflammatory properties of different extracts of Ligusticum chuanxiong. Their results demonstrated that the 95% ethanolic extract possessed the most potent anti-inflammatory activity. Bioactivity-guided investigation identified senkyunolide A and ligustilide as key constituents contributing to this effect. These compounds were shown to effectively attenuate inflammatory signaling by targeting multiple nodes, including extracellular signal-regulated kinase 2 (ERK2), COX-2, janus kinase 22, inhibitor kappa B kinase β, protein kinase C, and TNF-α, thereby modulating downstream proteins and exerting anti-inflammatory effects (78). In a related investigation, researchers purified two anti-neuroinflammatory compounds from *Ligusticum chuanxiong*. Chromatographic and spectroscopic analyses identified these compounds as senkyunolide A and Z-ligustilide. Both compounds significantly suppressed the production of pro-inflammatory mediators in lipopolysaccharide (LPS)-stimulated mouse BV-2 microglial cells and in human peripheral blood mononuclear cell-derived macrophages (79).

Senkyunolides exhibit significant anti-inflammatory and anti-migraine effects. Studies have shown that senkyunolide components could inhibit inflammatory responses by downregulating the levels of inflammatory factors, suppressing the NF-κB pathway, and thereby modulating downstream protein expression (80). Senkyunolide I significantly reduced the production of key inflammatory cytokines, including TNF-α, interleukin-6 (IL-6), and IL-1β (81, 82), supporting its immunomodulatory role. The underlying anti-inflammatory mechanism appears to involve two complementary pathways: upregulation of heat shock protein 70 in a heat shock factor-1-dependent manner, and inhibition of the toll like receptor 4 (TLR4)/NF-κB signaling cascade (83). Furthermore, at specific doses, both senkyunolide H and senkyunolide A significantly downregulated the mRNA expression of key components in the NF-κB signaling pathway, indicating that their anti-inflammatory activity involves suppression of this central inflammatory cascade—a mechanism linked to alterations in glycerophospholipid metabolism (84).

The MAPK signaling pathway plays a central role in inflammation by transducing extracellular stimuli into intracellular inflammatory responses. Its three principal subgroups—c-Jun N-terminal kinases (JNK), ERK, and p38 MAPK—serve as key mediators in this process (14). In microglial cells, senkyunolide H suppressed activation and alleviated LPS-induced neuroinflammation and oxidative stress through modulation of both the ERK and NF-κB pathways (85). Similarly, senkyunolide I has been shown to counteract elevated phosphorylation levels of JNK, ERK, p38, and p65 in murine lung tissue, further supporting the involvement of MAPK signaling in its anti-inflammatory action (86). Senkyunolide H moderately suppressed inhibitor of NF-κB α (IκBα) phosphorylation through the NF-κB pathway, and significantly inhibited JNK activation and ERK phosphorylation, thereby inhibiting osteoclast formation (87). Together, these mechanistic insights consolidate the evidence that senkyunolides exhibit substantial anti-inflammatory properties.

### 4.5 Antioxidant stress and analgesic effects

Oxidative stress plays a significant role in both the initiation and chronicification of migraine, and it interacts closely with the inflammatory process. Existing studies have demonstrated that migraine patients exhibit elevated levels of oxidative stress, and common migraine triggers such as noise, sleep deprivation, and air pollution appear to exacerbate this imbalance (88). Oxidative stress contributes to migraine pathogenesis not only by directly damaging biological macromolecules, but also by functioning as a signaling molecule that activates nociceptive pathways, stimulates the release of pro-inflammatory mediators, and dysregulates neurotransmitter release—collectively promoting migraine initiation and progression (89). Consistent with this role, studies indicate that during migraine attacks, levels of oxidative stress markers such as malondialdehyde (MDA) and 4-hydroxynonenal are markedly elevated, whereas the activity of endogenous antioxidant enzymes including superoxide dismutase (SOD) is significantly reduced (90).

Free radicals and reactive oxygen species (ROS) inflict damage on neurons and vascular endothelial cells, thereby exacerbating neuroinflammation and tissue injury. Excess ROS induce lipid peroxidation, generating cytotoxic MDA, while antioxidant enzymes—including SOD, glutathione peroxidase (GSH-Px), and reduced GSH—function to neutralize ROS. Under physiological conditions, nuclear factor erythroid 2-related factor 2 (Nrf2) is sequestered in the cytoplasm by its inhibitor Keap1. Upon oxidative challenge, Nrf2 dissociates from Keap1, translocates to the nucleus, and binds to the antioxidant response element (ARE), initiating the transcription of cytoprotective genes such as heme oxygenase-1 (HO-1), SOD, glutathione S-transferase, and NAD(P)H quinone oxidoreductase 1. Experimental studies demonstrated that senkyunolide I could reduce MDA levels, enhance SOD activity, and promote Nrf2 expression. Notably, treatment with senkyunolide I dose-dependently could increase the nuclear-to-cytoplasmic ratio of Nrf2. These results provide mechanistic evidence that senkyunolide I could alleviate oxidative stress primarily via activation of the Nrf2/ARE pathway (91). Senkyunolide I could attenuate ROS generation and disrupt the crosstalk between inflammation and oxidative stress. It concurrently enhanced the activity of key antioxidant enzymes, including catalase and GSH-Px, while suppressing the release of pro-inflammatory cytokines such as TNF-α, IL-1β, and IL-6. Moreover, senkyunolide I upregulated the expression of cytoprotective proteins like HO-1 and NQO1, and downregulated endoplasmic reticulum stress markers, including glucose-regulated protein 78 and C/EBP-homologous protein, collectively mitigating oxidative neuronal damage (92–94).

Senkyunolide H features a phthalide core bearing an allyl side chain, a structural motif that confers potent free-radical-scavenging capacity. *In vitro* studies confirmed its ability to inhibit lipid peroxidation. Furthermore, in rat pheochromocytoma PC12 cells, senkyunolide H prevented the loss of mitochondrial membrane potential (MMP) and suppressed the rise in ROS induced by the neurotoxin 1-methyl-4-phenylpyridinium. Together, these findings establish the antioxidant properties of senkyunolide H and support its role in protecting neural cells against oxidative stress-mediated damage (95).

CSD can lead to the upregulation of numerous genes, such as COX-2, TNF-α, IL-1β, galanin, and metalloproteinases (61). Astrocytes are critically involved in both the initiation and propagation of CSD. During CSD events, astrocytic calcium waves arise nearly synchronously with neuronal depolarization, and the astrocytic syncytium can further modulate CSD spread by regulating extracellular potassium and glutamate homeostasis. Notably, senkyunolide I has been shown to suppress LPS-induced astrocytic overactivation, enhance Nrf2 expression, and reduce the secretion of oxidative stress mediators in astrocytes, thereby normalizing astrocyte function (96).

Tianshu Capsule is prepared from the rhizomes of *Ligusticum chuanxiong* and *Gastrodia elata* in a 4:1 ratio, using a combination of ethanol extraction and water decoction. Preclinical studies in a nitroglycerin-induced migraine model using Sprague-Dawley rats demonstrated that Tianshu Capsule effectively modulated oxidative stress markers. It enhanced SOD activity and GSH levels while reducing MDA content. These antioxidant effects contribute to its efficacy in alleviating migraine-related pathology (97). Huifang Gao et al. conducted a time-dependent process study by identifying *in vivo* compounds in the blood of healthy rats and migraine rats after oral administration of Tianshu Capsule, gastrodin, ferulic acid, senkyunolide G, and senkyunolide I as representative compounds. Pharmacokinetic parameters demonstrated that the four compounds were rapidly absorbed into the bloodstream, with senkyunolide I also rapidly crossing the BBB into the brain (98). In behavioral assays, senkyunolide I exhibited significant analgesic activity. It prolonged the pain threshold in the hot-plate test and inhibited acetic acid-induced abdominal writhing responses in mice (40). Specifically, doses of 16 and 32 mg/kg significantly elevated the hot-plate pain threshold, and the 32 mg/kg dose also reduced the number of writhing episodes (41).

### 4.6 Improving BBB integrity

The integrity of the BBB is critically implicated in the pathogenesis of migraine. Migraine attacks are associated with transient impairment of BBB function, whereas pre-existing BBB dysfunction may itself serve as both a trigger for acute episodes and a perpetuating factor in the transition to chronic migraine. During migraine, particularly in cases with aura, pro-inflammatory cytokines such as TNF-α and IL-1β are released. These mediators can activate downstream inflammatory signaling cascades, which in turn promote BBB disruption, thereby establishing a vicious cycle that exacerbates neurovascular dysfunction (99). BBB disruption may permit peripheral immune cells to transmigrate into the central nervous system, thereby triggering neuroinflammation and aggravating migraine symptom (100). Additionally, BBB dysfunction may impair the regulation of neurotransmitters, which plays a key role in the pathogenesis of migraine (99).

As core active components of the traditional Chinese medicine Chuanxiong, senkyunolides have been confirmed by modern research to alleviate migraines through mechanisms closely associated with the protection and restoration of BBB function. Studies found that senkyunolide A, senkyunolide H, and senkyunolide I significantly increased the apparent permeability coefficient (Papp) of sodium fluorescein (Na-F). Notably, at equivalent concentrations, senkyunolide I exhibited the most pronounced effect on enhancing permeability in the bEnd.3 monoculture BBB model. It was further shown to modulate the expression of key tight-junction proteins: following treatment, protein levels of Occludin and zonula occludens-1 (ZO-1) in bEnd.3 cells underwent a transient decrease, returning to baseline within 8 h (101). Furthermore, under oxygen–glucose deprivation/reperfusion conditions, senkyunolide I downregulated both the protein and mRNA expression of the tight-junction proteins ZO-1 and Claudin-5 in an BBB spheroid model (102). In a separate *in vivo* context, it also conferred BBB protection in rats with sepsis-associated encephalopathy by downregulating matrix metallopeptidase 9 and aquaporin-4 expression, while upregulating levels of Nrf2 and occludin (103). Collectively, these regulatory actions help preserve BBB integrity, limit the influx of neurotoxic substances into the central nervous system, and consequently mitigate migraine-related pathology. The principal senkyunolides implicated in migraine treatment are summarized in Table 2.

**Table 2:** Senkyunolides for migraine headaches treatment.

Compounds	Pharmacological effects	Method	Model	Method of administration and dosage	Targets and details	Reference
Senkyunolide I	Suppressing activation of the trigeminal vascular system	*In vivo*	Nitroglycerin-induced Sprague–Dawley rats	Gavage administration: 72 mg/kg; intravenous administration: 20 mg/kg	Decreasing NO, β-EP, and plasma CGRP levels in rats, with no effect on NO or CGRP levels in brain tissue.	(40)
Senkyunolide I	Suppressing activation of the trigeminal vascular system	*In vivo*	Nitroglycerin induced Sprague–Dawley rats	Gavage administration: 18 mg/kg, 36 mg/kg, 72 mg/kg	Decreasing NO levels in rat plasma and whole brain tissue.	(41)
Senkyunolide I	Suppressing activation of the trigeminal vascular system	*In vivo*	Potassium chloride-induced CSD, CSD Sprague–Dawley rats	Intraperitoneal administration: 36 mg/kg, 72 mg/kg, 144 mg/kg	Decreasing NO and CGRP levels.	(43)
Senkyunolide I	Modulating pathological vascular Alterations	*In vivo*	Potassium chloride-induced CSD, CSD Sprague–Dawley rats	Intraperitoneal administration: 36 mg/kg, 72 mg/kg, 144 mg/kg	Significantly suppressed the amplitude of CSD.	(43)
Senkyunolide I	Modulating pathological vascular Alterations	*In vivo*	Nitroglycerin-induced Sprague–Dawley rats	Gavage administration: 18 mg/kg, 36mg/kg, 72 mg/kg	Decreasing NO levels in plasma and brain tissue.	(41)
Senkyunolide I	Modulating neurotransmitters and receptors	*In vivo*	Nitroglycerin-induced Sprague–Dawley rats	Gavage administration: 72 mg/kg;Intravenous administration:20 mg/kg	Decreasing NO, β-EP, and plasma CGRP levels in rats, with no effect on NO or CGRP levels in brain tissue.	(40)
Senkyunolide I	Modulating neurotransmitters and receptors	*In vivo*	Nitroglycerin-induced Sprague–Dawley rats	Gavage administration: 18 mg/kg, 36 mg/kg, 72 mg/kg	Regulating the levels and turnover rate of the monoamine neurotransmitter 5-HT in the brain, reducing the concentrations of NO and CGRP in plasma and brain tissue.	(41)
Senkyunolide I	Antioxidant stress	*In vitro*	LPS-induced BV2 cells	20 μmol/L, 50 μmol/L, 100 μmol/L	Inhibiting LPS-induced astrocyte hyperactivation, upregulating Nrf2 expression, reducing MDA and NO levels, increasing SOD activity, and decreasing the secretion of oxidative stress products by astrocytes.	(96)
Senkyunolide I	Analgesic	*In vivo*	Hot-plate test, acetic-acid-induced writhing test induced Kunming mice	Gavage administration: 72 mg/kg; intravenous administration: 20 mg/kg	Lengthening the thermal pain threshold in mice and suppressing acetic acid-induced writhing response.	(40)
Senkyunolide I	Analgesic	*In vivo*	Hot-plate test, acetic-acid-induced writhing test induced Kunming mice	Gavage administration: 8 mg/kg, 16 mg/kg, 32 mg/kg	Significantly elevating the pain threshold in mice and reducing the number of acetic acid-induced abdominal writhing responses.	(41)
senkyunolide A	Modulating pathological vascular alterations	*In vitro*	Isolated aorta induced by the prostanoid TP receptor agonist U46619 (10 and 30 nM for endothelium-removed and intact preparations, respectively), the-adrenoceptor agonist phenylephrine (0.1 and 3 μM for endothelium-removed and intact preparations, respectively), the 5-HT receptor agonist (5-HT, 10 μM) or the membrane depolarizing agent KCl (40 mM)	1–300 μM	Regulating ion channels and endothelial function to relax spastic blood vessels, restoring normal vascular tone and blood supply, improving circulation, and alleviating neurological symptoms caused by ischemia or hypoxia.	(55)
Senkyunolide A	Inhibiting inflammatory response	*In vitro*	LPS-induced BV2 cells	25, 50 μg/ml	Effectively inhibiting the expression of TNF-α and iNOS mRNA.	(79)
Senkyunolide H	Inhibiting inflammatory response	*In vitro*	LPS-induced BV2 cells	25μM, 50μM, 100μM	Reversing LPS-mediated microglial activation, neuroinflammation, and BV2 oxidative stress by modulating ERK and NF-κB pathways.	(85)
The ethyl acetate fraction of *Ligusticum chuanxiong* (including Senkyunolide I, senkyunolide H, and senkyunolide A)	Suppressing activation of the trigeminal vascular system	*In vivo*	Nitroglycerin-induced Sprague–Dawley rats	Gavage administration: 33 mg/kg	Significantly decreasing the levels of CGRP in rat plasma	(44)
CRCR (including senkyunolide A)	Suppressing activation of the trigeminal vascular system	*In vivo*	Nitroglycerin-induced Sprague–Dawley rats	Gavage administration: 4.4 g/kg	Significantly increasing cerebral blood flow in Nitroglycerin-induced migraine rats, reducing levels of ET-1, GABA, and NOS, increasing levels of 5-HT, 5-HIAA, and β-EP, and significantly downregulating CGRP levels and CGRP mRNA expression in the brainstem, as well as c-Fos mRNA expression.	(39)
XMT (including senkyunolide A)	Suppressing activation of the trigeminal vascular system	Clinical research	Migraine sufferers	Control group: conventional western medication; observation group: oral XMT (administered twice daily).	Significantly reducing the frequency and duration of headache episodes, regulating serum NO, CGRP, and ET levels, and decreasing the incidence of adverse reactions.	(46)
Extract of XMT	Suppressing activation of the trigeminal vascular system	*In vivo*	Dura catheterization surgery	Gavage administration: XMT *n*-BuOH—L(16.9 mg/kg), XMT *n*-BuOH—H (152.1 mg/kg), XMT EtOAc—L (41.6 mg/kg), XMT EtOAc—H (374.4 mg/kg), XMT *n*-BuOH + EtOAc—L (58.5 mg/kg), and XMT *n*-BuOH + EtOAc—H (526.5 mg/kg)	Modulating the expression of trigeminal migraine-related factors, positive expression, mRNA expression, protein expression of TRPV1, CGRP, CGRP-like receptor, and receptor activity-modifying protein by regulating the TRPV1-CGRP/CGRP-R pathway.	(14)
Shunaoxin pills (including senkyunolide I)	Modulating pathological vascular alterations	*In vitro*	Norepinephrine-induced and KCl-induced tonic contractions in endothelium-intact and -denuded rat aortic rings	0.625 mg/ml, 1.25 mg/ml and 1.875 mg/ml	Inducing less endothelium-dependent and more endothelium-dependent vasorelaxation through NO/cGMP and HO/CO pathways.	(57)
Suxiao Jiuxin Pill (including senkyunolide I and senkyunolide A)	Modulating pathological vascular alterations	*In vitro*	20 mM caffeine, 1 mmol/L phenylephrine, 20 mmol/L KCl-induced Human embryonic kidney 293 (HEK 293) cells; rat cardiomyoblast cells (H9C2 cells)	10 μmol/L	Exerting an inhibition effect mostly on ryanodine receptors and partly on voltage dependent calcium channels.	(59)
*Chuanxiong Rhizoma* essential oil	Modulating neurotransmitters and receptors	*In vivo*	Nitroglycerin-induced Sprague–Dawley rats	Gavage administration: 45 mg/kg, 90 mg/kg, 135 mg/kg	Improving migraine symptoms by elevating plasma 5-HT levels and enhancing 5-HT_1B_ protein expression in the periaqueductal gray region.	(70)
CRCR (including senkyunolide A)	Modulating neurotransmitters and receptors	*In vivo*	Nitroglycerin-induced Sprague–Dawley rats	Gavage administration: 4.4 g/kg, 6.6 g/kg	Increasing cerebral blood flow, reducing CGRP and c-Fos mRNA expression, and regulating the release of ET-1, GABA, NOS, 5-HT, 5-HIAA, CGRP, and β-EP in serum and the brainstem.	(39)
CRCR dropping pills (including senkyunolide I (2.88%), senkyunolide A(2.17%))	Modulating neurotransmitters and receptors	*In vivo*	Nitroglycerin-induced Sprague–Dawley rats	Gavage administration: 1.0 g/(kg·d), 1.5 g/(kg·d), 3.0 g/(kg·d)	Increasing 5-HT and β-EP levels in the hypothalamus of migraine model rats, simultaneously reducing plasma levels of CGRP and ET-1.	(72)
Tianshu capsule (including senkyunolide G, and senkyunolide I)	Antioxidant stress	*In vivo*	Nitroglycerin-induced Sprague–Dawley rats	150 mg/kg/day	Reducing oxidative stress markers (SOD, MDA, GSH).	(97)
*Ligusticum chuanxiong* (including senkyunolide A, senkyunolide H, and senkyunolide I)	Improving BBB integrity	*In vitro*	Transwell chamber constructed bEnd.3 cell *in vitro* BBB model	1 μmol/L, 10 μmol/L, 100 μmol/L	Regulating the expression of tight junction proteins in the BBB, reducing the expression levels of occludin and ZO-1 proteins.	(101)

## 5 Pharmacokinetic and stability studies of senkyunolides

### 5.1 Pharmacokinetic studies

Drawing on serum pharmacochemistry, Ying et al. analyzed the prototype constituents migrating into the blood and cerebrospinal fluid following administration of Chuanxiong's active fractions. Their study identified senkyunolide I as a component that enters both the systemic circulation and the brain, suggesting it may serve as the key bioactive constituent underlying the anti-migraine effects of these fractions (104).

As principal active constituents of Apiaceae medicinal plants such as *Ligusticum chuanxiong*, senkyunolides have been widely investigated for their pharmacokinetics, tissue distribution, and metabolism using advanced analytical techniques and experimental models. Liquid chromatography–mass spectrometry (LC-MS) has emerged as an essential analytical tool for quantifying senkyunolides due to its high sensitivity and specificity (105). For instance, an LC-MS method developed for the determination of senkyunolide I in rat plasma showed good linearity over the range of 6.75–675 μg/L, with a lower limit of detection of 6.75 μg/L, fulfilling the sensitivity requirements for pharmacokinetic studies (106). In addition, ultra-performance liquid chromatography/quadrupole-time-of-flight tandem mass spectrometry has been employed to rapidly profile senkyunolide I metabolites in rats after intravenous administration, revealing that methylation, hydration, epoxidation, glucuronidation, and glutathione conjugation represent its major metabolic pathways *in vivo* (107).

HPLC has likewise proven to be a robust analytical platform for senkyunolide quantification. A validated HPLC method for senkyunolide A demonstrated good linearity over a concentration range of 0.4–16.05 μg/ml, with intra- and inter-day precision (relative standard deviation < 5%) and a recovery rate exceeding 84%, meeting accepted criteria for bio-sample analysis (108). In a separate study, Junyong He et al. utilized HPLC to determine the concentration of senkyunolide I in plasma and tissues and applied non-compartmental analysis to derive pharmacokinetic parameters. Metabolites were further characterized by HPLC coupled with electrospray ionization tandem mass spectrometry. Their results showed that senkyunolide I was rapidly cleared from plasma following both oral and intravenous administration. The oral bioavailability was approximately 37.25%, which was markedly lower than the intraportal bioavailability (81.17%) but comparable to the intraduodenal bioavailability (36.91%). This pattern indicates a negligible gastric first-pass effect, whereas the hepatic first-pass effect accounted for about 18.83% of the observed reduction. After oral dosing in rats, senkyunolide I efficiently crossed the BBB and was widely distributed in peripheral tissues; the area under the concentration–time curve (AUC) ranked in the following order: kidney > liver > lung > muscle > brain > heart > thymus > spleen (109).

Intestinal absorption characteristics serve as a key determinant for the pharmacological efficacy of senkyunolides. For senkyunolide I, neither the absorption rate constant nor the Papp varied significantly across different concentrations (128.5–514 μg/ml) within the same intestinal segment, a profile consistent with passive diffusion as the primary absorption mechanism rather than carrier-mediated active transport (110). Notably, the absorption profiles of individual senkyunolide constituents within a Chuanxiong water decoction differ substantially from one another (111). Absorption efficiency also varies markedly along the intestinal tract: studies identify the duodenum as the optimal site for senkyunolide I absorption—a finding relevant to the design of oral delivery systems (110)—and report that the Papp of senkyunolide A is highest in the duodenum and lower in the jejunum and ileum (112).

Drug metabolism plays a critical role in governing the safety and efficacy of pharmaceutical agents, thereby informing the development and clinical translation of senkyunolide-based therapeutics. Senkyunolides are, in fact, important metabolites derived from ligustilide, undergoing biotransformation—catalyzed by NADPH-dependent monooxygenases—into compounds such as senkyunolide H and I (12). Like their precursor, senkyunolides themselves undergo extensive metabolic processing *in vivo*, a factor that directly governs their systemic bioavailability and resultant pharmacological activity.

Metabolic studies indicated that senkyunolide A undergoed a typical two-phase biotransformation *in vivo*, with metabolite profiles differing across biological matrices—a reflection of the complexity of its metabolic network. Similarly, senkyunolide I was subject to diverse metabolic modifications, including Phase I reactions (e.g., methylation, hydrolysis, epoxidation) and Phase II conjugations (e.g., glucuronidation, glutathione conjugation) (113–115). After oral administration of senkyunolide I (36 mg/kg), the parent compound and nine metabolites were identified in rat bile (109).

Following the oral administration of Chuanxiong essential oil (10 mL/kg, containing senkyunolide A at 26.4 mg/ml) in a 5% CMC-Na solution, Qinhui Wang et al. evaluated its pharmacokinetics by using a noncompartmental model. The results showed that the AUC_0−*t*_, AUC_0−∞_, t_1/2_, and C_max_ of senkyunolide A were 4,791 ± 156 mg/L·h, 5,748 ± 664 mg/L·h, 9.7 ± 3.3 h, and 874 ± 12 mg/L, respectively. Notably, co-administration of *Angelica dahurica* essential oil with Chuanxiong essential oil was observed to mitigate the systemic accumulation risk of constituents such as senkyunolide A, which may consequently lower potential toxicity. Furthermore, these pharmacokinetic alterations are likely attributable to modulations in absorption and metabolism mediated by factors including gut microbiota composition, endogenous biomolecules, enzyme activity, and transmembrane transport efficiency (116). A comparative LC-MS study was performed to evaluate the pharmacokinetics of senkyunolide I in rats following administration of a modified Huoluo Xiaoling Dan formulation vs. a single-herb Chuanxiong extract. Although plasma concentration–time profiles for both treatments fit a two-compartment model, significant differences were observed in key pharmacokinetic parameters of senkyunolide I between the compound prescription and the single-herb group. This suggests that other herbal constituents in the compound formulation may markedly influence the absorption of senkyunolide I, potentially by enhancing its dissolution, inhibiting intestinal metabolism, or modulating transport protein activity (106). A pharmacokinetic study of senkyunolide A in Buyang Huanwu Decoction and its derived formulation (Naojian Tablets) also revealed the holistic effects of compound compatibility. Using the total statistical moment method, the study integrated the overall pharmacokinetic parameters of three components—ferulic acid, senkyunolide A, and ligustilide—and found that the similarity index of the total parameters between Buyang Huanwu Decoction and Naojian Tablets reached as high as 0.9778. The AUC values of the compound preparations were 240.6 and 133.0 μg·h/ml, respectively, while the mean residence times were 3.192 min and 3.259 min, respectively (117). The combination of borneol (0.08 g/kg) with Chuanxiong injection significantly increased the AUC and C_max_ of senkyunolide I in brain tissues. The relative bioavailability increased by 1.8-fold in the cortex and 1.5-fold in the hippocampus (118). A comparative pharmacokinetic study in rats demonstrated that the oral administration of senkyunolide I and senkyunolide H resulted in significantly higher systemic exposure in nitroglycerin-induced migraine models compared to healthy controls. Specifically, the model group showed marked elevations in AUC_0−*t*_, AUC_0−∞_, and C_max_ for both compounds. In contrast, the t_1/2_ of both senkyunolide I and senkyunolide H was significantly shorter in the migraine model group. This accelerated elimination may be due to nitroglycerin- and NO-induced increases in microvascular permeability, which promote drug absorption and clearance. Furthermore, both senkyunolide I and H displayed a characteristic double-peak concentration–time profile *in vivo*, likely attributable to the metabolic interconversion of their precursor, ligustilide (119).

The route of administration critically governs drug absorption and disposition, making its investigation essential for advancing pharmacokinetic understanding. A comparative study of senkyunolide A (100 mg/kg) administered via oral and intravenous routes showed that after intravenous injection, its apparent volume of distribution was 6.74 ± 0.73 L/kg, clearance was 7.2 ± 0.48 L/h, and t1/_2_ was 0.65 ± 0.06 h, reflecting rapid and extensive tissue distribution accompanied by fast systemic clearance. Overall, senkyunolide A displays pharmacokinetic properties of quick absorption, broad distribution, and rapid elimination. However, its oral bioavailability (≈8%) was markedly lower than that observed after intravenous dosing (≈75%), indicating that approximately 67% of the orally administered dose is lost during gastrointestinal absorption (120).

### 5.2 Research on improving the properties of senkyunolides

Senkyunolides are prone to degradation under common environmental conditions. While senkyunolide I displays reasonable stability in weakly acidic media, its degradation accelerates markedly in alkaline environments. Molecular oxygen is a primary destabilizing factor for both senkyunolide A and I, with light and heat further promoting their breakdown under aerobic conditions (121). Long-term stability studies reveal distinct fates: after 2 months at ambient temperature under natural light, senkyunolide A converted into the more stable butylphthalide, whereas senkyunolide I—lacking a double bond in its six-membered ring—undergoed only slow, partial isomerization (122). Consistent with this inherent instability, senkyunolide I exhibited rapid distribution and elimination in rats following both intravenous and intragastric administration (40). These findings indicated that although senkyunolides had demonstrated efficacy in improving migraine symptoms, they shared several common limitations: structural instability and susceptibility to isomerization during preparation, poor water solubility, sensitivity to light and heat, variable oral absorption, and rapid elimination *in vivo*. Consequently, current research is actively focused on developing strategies to overcome these limitations through formulation and delivery innovations.

Cyclodextrin inclusion complexation is a supramolecular encapsulation strategy that employs cyclic oligosaccharides (cyclodextrins) as host molecules. Through non-covalent interactions, guest molecules—such as volatile oils—are incorporated into the hydrophobic cavity of the cyclodextrin, forming stable inclusion complexes. This approach effectively shields the encapsulated compounds from oxygen, light, and heat, thereby suppressing degradation pathways while concurrently improving their aqueous solubility. Li Chao et al. prepared inclusion complexes of Chuanxiong volatile oil using β-cyclodextrin (β-CD) and hydroxypropyl-β-cyclodextrin (HP-β-CD), concurrently quantifying ligustilide and senkyunolide A via HPLC. Their results indicated that increasing the cyclodextrin proportion markedly enhanced the stability of the volatile oil inclusion complexes, with the β-CD complex exhibiting superior stabilization. The optimal mass ratio was determined to be 1:8 (volatile oil: cyclodextrin), under which the predicted shelf life exceeded 4 years (123). Functionalized modifications offer dual advantages in terms of stability and solubilization. Yang Yuting et al. utilized HP-β-CD inclusion complexes to increase the solubility of ligustilide in Chuanxiong oil by 15.57-fold. Thermal acceleration tests demonstrated that after 10 days, the relative retention rates of ligustilide and senkyunolide A in the inclusion complexes (90.36%) were significantly higher than those in physical mixtures (43.03%) and free volatile oil (64.95%) (124). Volatile oils obtained from the rhizomes of Chuanxiong and Danggui are rich in senkyunolides, yet they exhibit limited water solubility and poor oral bioavailability. Microemulsions, which are drug delivery systems composed of an oil phase, an emulsifier, and an aqueous phase, have emerged as an effective drug delivery technology. Their high solubilizing capacity and nanoscale droplet size promote efficient permeation across gastrointestinal barriers, thereby addressing the delivery challenges associated with these volatile oils (125). Zhang et al. formulated a microemulsion system for the co-delivery of volatile oils from Chuanxiong and Danggui rhizomes. Compared to the unformulated oils, this microemulsion significantly enhanced oral bioavailability and potentiated anti-inflammatory activity (126). Separately, liposomes, vesicular carriers formed by the self-assembly of phospholipid bilayers, are well-suited for encapsulating lipophilic drugs due to their hydrophobic lipid core. To address the inherent instability of Chuanxiong essential oil, a stable liposomal formulation was developed. These CXEO-loaded liposomes have demonstrated favorable safety profiles along with anti-inflammatory and antioxidant properties (127).

Self-emulsifying drug delivery systems are isotropic mixtures of an oil phase, emulsifiers, and co-solvents that can exist as either liquids or solids. Owing to their excellent physical stability and fine droplet size upon dispersion, self-emulsifying drug delivery systems represent an advanced oral delivery platform compared to conventional emulsions. Qin et al. (128) developed a self-emulsifying drug delivery system formulation for Chuanxiong oil, which maintained excellent stability under both light-exposure and high-temperature stress conditions.

## 6 Conclusions and expectations

Senkyunolides serve as the principal metabolites of ligustilide, both *in vitro* and *in vivo*. While direct extraction from fresh medicinal plants such as *Ligusticum chuanxiong* yields only limited quantities of these compounds, studies indicate that the degradation of ligustilide during herb processing and storage results in substantially higher senkyunolide levels (114). Furthermore, metabolic investigations confirm that ligustilide is biotransformed *in vivo* into senkyunolide I, senkyunolide H, and butylidenephthalide. These lines of evidence strongly support the notion that senkyunolides significantly contribute to the pharmacological activities attributed to ligustilide in living systems (12, 129).

Senkyunolides, as the core active components of Apiaceae plants such as *Ligusticum chuanxiong*, exert anti-migraine effects through multi-target and multi-level mechanisms of action. This integrated regulatory characteristic enables them to intervene in the complex pathological network of migraine while reducing the risk of compensatory escape and drug resistance often associated with single-target therapies. From the Shennong Bencao Jing to modern compound formulations, the millennia of clinical use provide indirect clues to the real-world efficacy of the active constituent senkyunolides. This article provides a systematic review of the classification, extraction methods, anti-migraine mechanisms, pharmacokinetic properties, and stability improvement strategies of senkyunolides. The multi-dimensional homeostatic effects of senkyunolides on the neurovascular unit, as elaborated in this review, align closely with the pathophysiological network of migraine. Furthermore, the small molecular size, inherent blood-brain barrier permeability, and detectable presence in cerebrospinal fluid of senkyunolides collectively enhance their delivery to the central nervous system, underscoring their promise as a potential therapeutic agent for migraine. Importantly, all of this positive evidence currently stems from *in vitro* studies, animal models, and pharmacokinetic simulations, and lacks direct validation from human clinical trials.

Nevertheless, stability challenges continue to constrain their further development and clinical application. The inherent chemical lability of senkyunolides renders them susceptible to degradation by light, heat, and oxygen throughout processing, storage, and application. Future research must prioritize the development of more effective stabilization strategies, such as novel cyclodextrin derivatives, nanocarriers, and advanced liposomal systems, to enhance both their physicochemical stability and bioavailability. Parallel efforts in structural modification or the synthesis of stabilized analogs represent another crucial avenue for improving molecular durability. Currently, the extraction and formulation technologies for senkyunolides remain suboptimal, facing persistent hurdles including low extraction yields and inadequate formulation stability. Advancing efficient extraction protocols and innovative formulation platforms is essential for enhancing both the yield and stability of senkyunolides. Concurrently, capitalizing on non-oral delivery routes—such as nasal or transdermal administration—and refining dosing regimens are expected to broaden their therapeutic applicability. through exposure-matched trials. To address the lack of long-term safety data, future evaluations should necessitate comprehensive preclinical and clinical toxicological studies. These studies must establish a clear safety dosage window and identify target organ toxicity, with a dedicated focus on the risks of co-administration with medications for common migraine comorbidities (e.g., antidepressants and cardiovascular drugs). Furthermore, during the preclinical development of senkyunolide-based therapeutics, high-throughput screening technologies can be utilized to systematically profile their activity against common receptors, ion channels, and key enzyme systems, thereby predicting potential off-target pharmacological effects. With in-depth research on senkyunolides, particularly advances in clinical trials and the application of novel formulation technologies, this important active component is expected to become a valuable addition to the field of migraine treatment, bringing new hope to patients worldwide. Future studies should focus on addressing key challenges in clinical translation, accelerating the transformation of laboratory achievements into clinical applications, and ultimately benefiting patients. In summary, senkyunolide compounds show great potential as natural anti-migraine agents, but further exploration is needed regarding their mechanisms of action, stability improvement, formulation technologies, and clinical research. Through multidisciplinary collaboration and innovative research, their clinical translation process can be accelerated, providing migraine patients with more efficient and low-toxicity treatment options.
